# An openEHR based approach to improve the semantic interoperability of clinical data registry

**DOI:** 10.1186/s12911-018-0596-8

**Published:** 2018-03-22

**Authors:** Lingtong Min, Qi Tian, Xudong Lu, Jiye An, Huilong Duan

**Affiliations:** College of Biomedical Engineering and Instrument Science, Zhejiang University, Zheda Road, Hanghzou, 310027 China

**Keywords:** openEHR, Clinical data registry, Archetypes, Semantic interoperability

## Abstract

**Background:**

Clinical data registry is designed to collect and manage information about the practices and outcomes of a patient population for improving the quality and safety of care and facilitating novel researches. Semantic interoperability is a challenge when integrating the data from more than one clinical data registry. The openEHR approach can represent the information and knowledge semantics by multi-level modeling, and it advocates the use of collaborative modeling to facilitate reusing existing archetypes with consistent semantics so as to be a potential solution to improve the semantic interoperability.

**Methods:**

This paper proposed an openEHR based approach to improve the semantic interoperability of clinical data registry. The approach consists of five steps: clinical data registry meta-information collection, data element definition, archetype modeling, template editing, and implementation. Through collaborative modeling and maximum reusing of existing archetype at the archetype modeling step, the approach can improve semantic interoperability. To verify the feasibility of the approach, this paper conducted a case study of building a Coronary Computed Tomography Angiography (CCTA) registry that can interoperate with an existing Electronic Health Record (EHR) system.

**Results:**

The CCTA registry includes 183 data elements, which involves 20 archetypes. A total number of 45 CCTA data elements and EHR data elements have semantic overlap. Among them, 38 (84%) CCTA data elements can be found in the 10 reused EHR archetypes. These corresponding clinical data can be collected from the EHR system directly without transformation. The other 7 (16%) CCTA data elements correspond to one coarse-grained EHR data elements, and these clinical data can be collected with mapping rules. The results show that the approach can improve semantic interoperability of clinical data registry.

**Conclusions:**

Using an openEHR based approach to develop clinical data registry can improve the semantic interoperability. Meanwhile, some challenges for broader semantic interoperability are identified, including domain experts’ involvement, archetype sharing and reusing, and archetype semantic mapping. Collaborative modeling, easy-to-use tools, and semantic relationship establishment are potential solutions for these challenges. This study provides some experience and insight about clinical modeling and clinical data registry development.

## Background

Clinical data registry, also known as clinical registry, patient registry, disease registry, and outcomes registry, is increasingly being developed and used around the world. The National Institutes of Health describe registry as “a collection of information about individuals, usually focused on a specific diagnosis or condition.” [1]. National Quality Registry Network defines clinical registry as “a clinical registry records information about the health status of patients and the health care they receive over varying periods of time.” [2]. Gliklich RE et al. [3] define clinical registry as “an organized system that uses observational study methods to collect uniform data (clinical and other) to evaluate specified outcomes for a population defined by a particular disease, condition, or exposure and that serves predetermined scientific, clinical, or policy purpose(s).”. All of these definitions of clinical registry have one or more of the following features: (1) aiming at one or more objectives or purposes; (2) having a specific criteria to certify the eligibility of patient data; (3) providing a real-world health care view; (4) having a broader patient population.

The stakeholders of clinical registry include clinicians, health care organizations, patients, researchers, device or drug manufactures, etc. So far, a large number of clinical data registries have been developed for different goals, such as benchmarking and outcomes evaluation [4–7], drugs [8, 9], complications [10, 11], comparative effectiveness [12, 13], clinical treatments [14–17] and reports [18–21]. There are more than 3000 registries listed in the Registry of Patient Registries (RoPR) [22].

Data integration is necessary when some studies are conducted based on a broader population across various clinical registries and other data sources. As most clinical data registries are developed and maintained by specific vendors based on various private information models. The private information model has two major problems: (1) the same data element has different names; (2) the same data element name has different definitions. Without semantic interoperability, the data integration between two different clinical data registries will be a tremendous workload, which hinders the data utilization of clinical data registries. Given that collaborations are conducted between clinical data registries, semantic interoperability is a challenge for clinical data registry.

The openEHR community initiated its approach with a set of specifications published by the openEHR Foundation [23]. The openEHR approach can improve semantic interoperability [24, 25]. It is a multi-level modeling approach, and has already been implemented in several countries (e.g. United Kingdom, Australia, and China, etc.) [25–37]. The multi-level model consists of a Reference Model (RM), archetypes and templates. RM is a formal and stable information model that defines logical structures of health information, to support the syntactic interoperability. Archetypes enable both syntactic interoperability and semantic interpretability, which are two necessary components of semantic interoperability. An extra benefit is that archetypes can be defined and understood by domain experts. Another important aspect of semantic interoperability is archetype reusing by collaborative modeling. In this regard,several tools and platforms supporting the archetype collaborative modeling have been developed.

The openEHR approach provides the basis for semantic interoperability via the multi-level modeling. However, it lacks specific solutions for developing openEHR based clinical data registries. To address this challenge, this study develops a novel approach to boost the semantic interoperability between clinical data registries. A case study using maximum reuse of existing archetypes is presented to validate the feasibility of our proposed approach.

## Methods

### Approach description

**Fig. 1:**
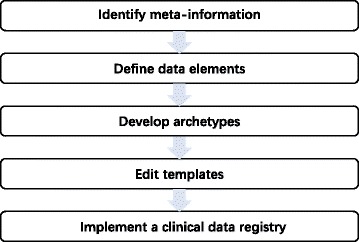
The flowchart of an openEHR based approach to develop clinical data registry

Combining the openEHR approach and clinical data registry user’ guide [3], we proposed a five-step openEHR based approach to develop clinical data registry, including meta-information identification, data elements definition, archetypes modeling, templates editing, and clinical registry implementation. The flowchart of the approach is shown in Fig. 1.

#### Identify meta-information of clinical registry

In this step, we designed a questionnaire to collect the meta-information from the owner and users of the clinical data registry. The questionnaire contains the purposes, key stakeholders, governance, data scope, and target population of the clinical data registry. The meta-information affect the choice of the registry type, the data elements to be captured, the process of data collection, and the patterns of data utilization [3].

#### Define data elements

Then the data elements were defined by the stakeholders of a clinical registry. They can be recorded in paper and electronic format. The data elements determine the scope, structure, and granularity of data collection. They are the representation of data requirements of clinical data registry and the resource for archetype modeling. The definition of each data element includes data elements name, data type, description, and value set.

#### Develop archetypes

**Fig. 2:**
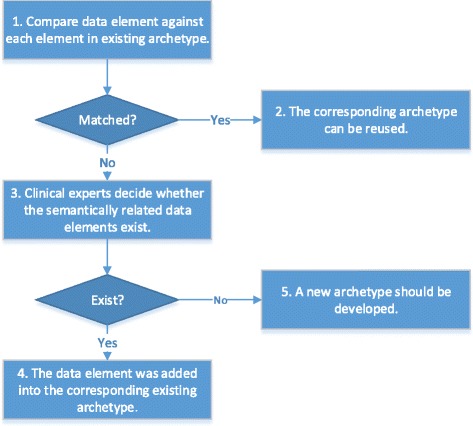
The flowchart of the archetype modeling method

The process of developing archetypes was illustrated in Fig. 2.

1. The one who is responsible for archetypes retrieving compares data element against each element in existing archetypes. This work can be done manually, or with the tools, such as Clinical Knowledge Manager(CKM) [38].

2. If a match was found, the corresponding archetype can be reused.

3. If a match was not found, clinical experts decided whether the semantically related data elements exist.

4. If the semantically related data elements were found, the data element was added into the corresponding existing archetype.

5. If the semantically related data elements were not be found, a new archetype should be developed.

Stakeholders, especially clinical experts, were encouraged to involve in the process of new archetype developing, considering that they may be ultimate users of the clinical data and know the semantics of clinical data.

**Fig. 3:**
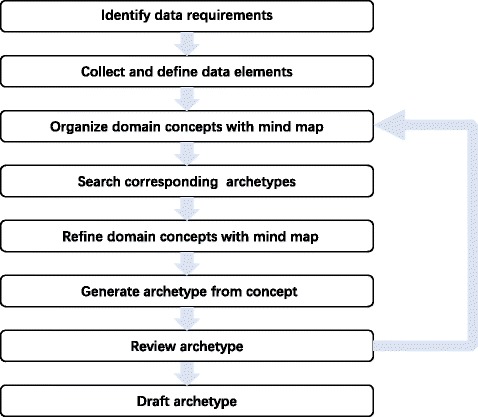
The flowchart of new archetype developing

While developing new archetypes, some pinciples [39], archetype modeling methods [30, 35, 40, 41] and tools [42–44] are provided to guide and assist archetype modeling. Yet, clinical experts cannot participate in the modeling process easily. To facilitate clinical experts playing a leading role in archetype modeling, we proposed a new archetype modeling method and developed an online archetype modeling tool named Domain-involved Archetype Editor(DiAE). DiAE generates archetypes from mind maps based on mapping rules. This modeling method is an iterative process and consists of identifying data requirements, collecting data elements, organizing clinical concepts with mind maps, mapping clinical concepts into archetypes, and reviewing archetypes, which is shown in Fig. 3. In this method, mind maps are essential for the communication among stakeholders. By using DiAE, the stakeholders can define the data elements, and develop archetypes semi-automatically through editing mind maps with the drag-and-drop editing function. Archetypes can be generated from concepts confirmed by clinical experts through the archetype mapping module of DiAE. The archetype mapping module was developed based on rules of relationship mapping between clinical concept and archetype.

Without necessary coordination mechanisms, different modelers may build entirely different archetypes. Collaborative modeling can facilitate archetypes unified development and sharing. We used Health Archetype Collaboration (HAC) [45] facilitate the consistency of archetyping for clinical data registries, which is an online archetype collaborative modeling platform.

To improve the quality of archetypes, we organized experts panels to review archetypes based on HAC. The scope of review comprised three parts: meta-data review; content review; terminologies review. As clinical experts lack archetype knowledge and information technology knowledge, we used the mind map as the communication media during the process of review. The meta-data review covered concept name, description, use, misuse, keywords, and purpose of archetypes. The content review covered data item name, data item description, and logical relationship between elements. The terminologies review focuses on the reference and constraints of terminologies. If clinical experts approved the archetype, we would apply it into CCTA registry. Otherwise, this archetype would enter a new round of iterative process until it passes the review.

#### Edit templates

Stakeholders edited templates with the template editing tools by reducing undesired data elements of archetypes and adding the required configuration information.Implementers usually develop them according to local requirements. The templates will be used to generate data storage structure, application program interfaces (APIs), and user interfaces (UIs).

#### Implement clinical data registry

After developing archetypes and templates, the data semantics of clinical registry was expressed completely. To achieve semantic interoperability, the RM was implemented as a software module, and the clinical data registry corresponding templates were consumed within the software module. The template-generated artifacts include data storage structure, APIs, and UIs.

### Case design and verification

We conducted a case study to verify the feasibility of improving semantic interoperability based on this clinical registry development approach. First, we developed a CCTA registry. Then we collected specific clinical data into the CCTA registry from an existing EHR system.

#### Background of CCTA registry

The CCTA data registry is included in one of a National Key R&D Program, which is established to promote early detection and warning of coronary atherosclerosis. The CCTA data registry covers clinical information and CCTA imaging examination information. The coronary disease experts were in charge of data elements definition. The CCTA registry was designed to collect clinical data of patients who have undergone CCTA examination. Clinicians and health data analysis experts are the end users.

#### The development of CCTA registry

We initially organized an expert panel to identify the meta-information of CCTA registry and divided data requirements into four categories: patient demographic data, baseline data, CCTA parameter data, CCTA report data. Then, clinical experts defined data elements with DiAE, including name, description, terminology or constraints, data type, and reference source.

Based on these data elements, clinical experts initially drew clinical concepts in the form of mind map using the drag-and-drop function of DiAE. To improve semantic interoperability of CCTA registry, we reused existing archetypes. Given that the CCTA registry needed to extract specific clinical data from an EHR system, we developed archetypes based on the aforementioned approach through reusing existing archetypes implemented within the EHR system and CKM. If corresponding archetypes appeared in both EHR archetypes repository and CKM, we reused the EHR archetypes. If the corresponding archetypes only appeared in CKM, then we reused the existing CKM archetypes.

We edited templates according to the concrete requirements of the CCTA registry and built the CCTA registry by implementing them based on the relational database mapping (ARM) [37].

#### Specific clinical data collection from an existing EHR system

After developing CCTA registry, we extracted specific clinical data from the EHR system by invoking the APIs generated according to corresponding archetypes. The specific clinical data includes patient demographic data, diagnosis data, admission data, medication data, imaging examination data, and physical sign data.

## Results

For CCTA registry, clinical experts defined 183 data elements, and 20 archetypes (Table 1), and ten templates. Among these archetypes, there were 17 reused archetypes. Among them 10 archetypes were from EHR archetype repository, 7 archetypes were from CKM. Beside these reused archetypes, 3 new archetypes were developed by clinical experts. 10 relational database tables were generated from these archetypes and templates. The data collected from the EHR system consists of demographics, diagnosis data, admission data, physical sign data, image examination data.

**Table 1:** Archetypes of CCTA clinical data registry

Reused from EHR archetypes	Reused from CKM	New
DEM-PARTY_IDENTITY. person_name.v1	OBS.blood_pressure.v1	OBS.exercise_summary.v1
DEM-PERSON.person-patient.v1	EVA.tobacco_smoking_summary.v1	OBS.CCTA_parameter.v1
DEM-ITEM_TREE.person.v1_details.v1	OBS.alcohol_use.v1	OBS.CCTA_report.v1
DEM-ADDRESS.address.v1	EVA.family_history.v2	
DEM-ADDRESS.electronic_communication.v1	OBS.body_weight.v2	
ADM_ENTRY.admission.v1	OBS.height.v1	
EVA.problem_diagnosis.v1	OBS.pulse.v1	
INS.medication_order.v1		
INS.request-imaging_exam.v1		
CLU.contact.v1		

DEM is short for openEHR-DEMOGRAPHIC; OBS is short for openEHR-OBSERVATION; INS is short for openEHR-INSTRUCTION; CLU is short for openEHR-CLUSTER; ADM is short for openEHR-ADMIN_ENTRY

Clinical experts are willing to use mind maps as the communication media during the archetype modeling process. For CCTA archetype modeling, clinical experts play a dominant role throughout the entire process, especially data elements collection, clinical concepts definition, and archetypes review. They consider the collaborative archetype modeling as a good mode that can improve semantic interoperability.

The archetypes reused directly include 5 demographic information related archetypes, 2 admission related archetypes, one diagnosis related archetype, one imaging examination archetype, one alcohol use summary archetype, one medication-related archetype, and four physical signs related archetypes.

The modified archetypes include a smoking summary archetype and a family history archetype. As the representation of the type and unit are different, we added new data items to record the specific content within “openEHR-EHR-EVALUATION.tobacco_smoking_summary.v1” and “op-enEHR-EHR-EVALUAT-ION.family_history.v2”.

We defined three new archetypes to represent the CCTA examination parameters, CCTA report, and physical exercise summary information.

Supported by the archetype modeling method proposed and the DiAE, clinical experts can involve in archetype modeling. But it is not easy to develop high-quality archetypes by clinical experts independently, since archetype modeling requires clinical knowledge, archetype knowledge, and technological knowledge simultaneously.

A total number of 45 CCTA data elements and EHR data elements have semantic overlap. Among them, 38 (84%) CCTA data elements can be found in the 10 reused EHR archetypes. These corresponding clinical data can be collected from the EHR system directly without transformation. The other 7 (16%) CCTA data elements corresponding to one coarse-grained EHR data elements, and these clinical data can be collected with mapping rules.

## Discussion

The openEHR based approach to develop clinical data registry can improve the semantic interoperability through reusing existing archetypes. On one hand, RM represents the information semantics and supports syntactic interoperability. On the other hand, archetypes and templates represent the domain semantics and enable semantic interoperability. In this study, CCTA archetypes were developed by reusing existing archetypes of an EHR system, and the specific clinical data of EHR system can be reused by CCTA registry.

The formal and sharable archetypes are the premise of improving the semantic interoperability of health information system built with the openEHR based approach. If the existing archetypes are reused without modification, the corresponding health information can be exchanged and shared without transformation. If existing archetypes are reused with some modifications, the health information represented by the common elements of them can be understood and computed. In this study, some added data elements in CCTA archetypes have finer granularity than corresponding EHR data elements, and the corresponding clinical data cannot be collected from the EHR system directly.

Although there are hundreds of archetypes stored in several archetype repositories, it is inevitable to develop new archetypes to follow the evolving health knowledge. Given that archetypes represent the formal definitions of clinical content and express the health knowledge, clinical domain experts should involve in archetype developing and play important roles. Considering archetype is a kind of information artifact that can be computed by information technology, the opinions of information domain experts should not be omitted during the archetype developing process. How to make domain experts involve in archetype modeling is a challenge [46]. In this study, domain experts can take part in archetype modeling through drawing mind maps for generating archetypes with DiAE.

Broader archetype sharing and reusing would facilitate semantic interoperability. However, a broader archetype sharing and reusing is a challenge. Collaborative modeling can respond to this challenge by developing a set of formal archetypes. Collaborative modeling aims to utilize relevant resources to develop, manage, and share consistent archetypes. The resources that collaborative modeling can utilize include clinical experts, information experts, vendors, researchers, hospitals, and other archetype consumers. They play diverse roles, including requirements and/or knowledge provider, archetype developer, archetype manager, archetype reviewer, and archetype consumers. Based on the same storage, unified management, and open tools, diverse roles give specific contributions to develop and share formal archetypes.

Although the development of archetypes is constrained by RM and ADL, it is too flexible to keep a consistent structure among different developers. An archetype is constrained by the archetype type, data structure, data type defined in RM. Each archetype should include six components: archetype identification, concept, language, description, definition, and ontology. Different developers may develop various archetypes for the same clinical concept, which will hinder the semantic interoperability. The sharable archetype design pattern will be useful to guide various developers to develop sharable archetypes. These design patterns are the archetypes that define the basic content and structure of general concepts. They are a synthesis of mature experience and knowledge of archetype modeling. Based on the same design pattern, it is easier to develop formal archetype with consistent semantics.

Although reusing existing archetypes is an important principle of archetype development, some semantic granularity modifications are inevitable during the modeling process. Semantic mapping among different granularity data elements or archetypes is of great significance for semantic interoperability. In this study, the granularity of physical sign information is different in EHR archetypes and CCTA archetypes. Among EHR archetypes, there is a general archetype to represent all the physical sign information. While CCTA registry uses several fine-grained archetypes to represent specific kinds of physical sign information, including blood pressure, heartbeat, height, and weight. Although the general EHR physical sign archetype and these CCTA specific physical sign archetypes should have a semantic relationship, semantic interoperability between them cannot be achieved due to the lack of semantic relationship representation among these archetypes.

The process of archetype modeling for CCTA requirements provides experience and best practices for clinical information modeling. These archetypes will serve as a reference for formulating CCTA-related data standard, including data item and terminologies.

## Conclusion

In this study, we proposed an openEHR based approach to develop clinical data registry for improving the semantic interoperability and verified its feasibility through the CCTA registry case study. This study also provides experience and insights about clinical modeling and clinical registry development. Although reusing archetypes can improve semantic interoperability, there are some challenges hindering the broader semantic interoperability, including domain experts’ involvements, archetype sharing and reusing, and archetype semantic mapping, etc. Collaborative modeling and easy-to-use tools can respond to these challenges. The establishment of a semantic relationship between archetypes can promote archetype semantic mapping. We will address these challenges in our future work.

## Abbreviations

APIs: Application programming interfaces
ADL: Archetype definition language
CCTA: Coronary computed tomography angiography
CKM: Clinical knowledge manager
DiAE: Domain-involved archetype editor
EHR: Electronic health record
HAC: Health archetype collaboration
RM: Reference model
RoPR: Registry of patient registries
UIs: User interfaces
